# Supplementary material for: Machine Friendly Machine Learning: Interpretation of Computed Tomography Without Image Reconstruction
Source: Sci Rep. 2019 Oct 29;9:15540. doi: 10.1038/s41598-019-51779-5 (PMC6820559; doi:10.1038/s41598-019-51779-5)
Supplement: Supplementary file 1 — Supplementary Information [file 41598_2019_51779_MOESM1_ESM.pdf]

# Machine Friendly Machine Learning: Interpretation of Computed Tomography Without Image Reconstruction

Hyunkwang Lee[1,2], Chao Huang[1], Sehyo Yune[1], Shahein H. Tajmir[1], Myeongchan Kim[1], and Synho Do[1,*]

[1]Department of Radiology, Massachusetts General Hospital, Boston, MA 02114, USA
[2]John A. Paulson School of Engineering and Applied Sciences, Harvard University, Cambridge, MA 02138, USA
[*]sdo@mgh.harvard.edu

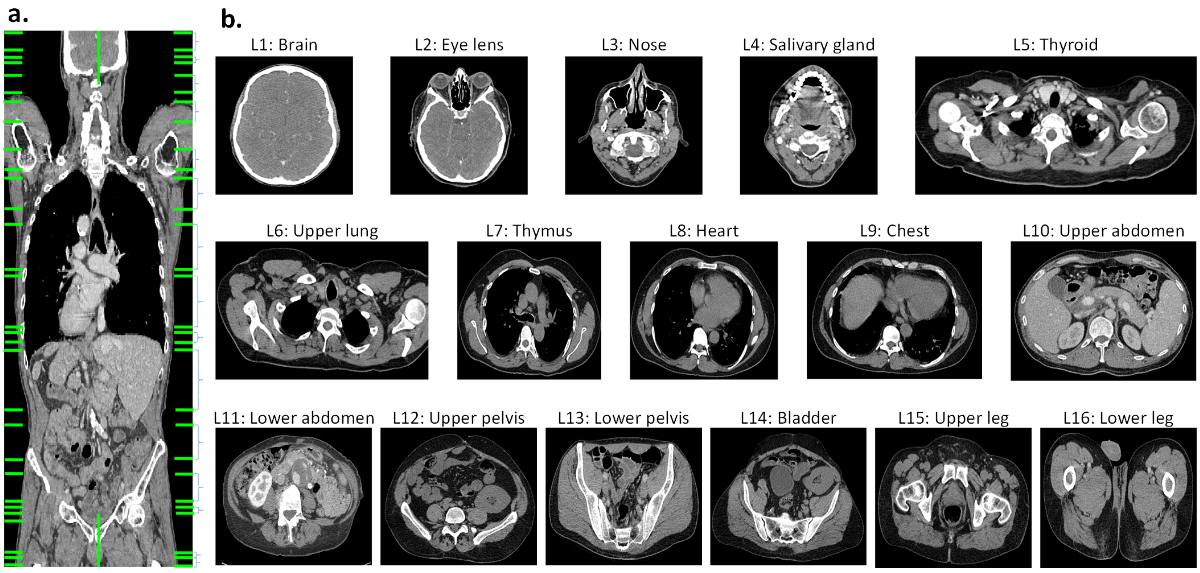


**Figure S1.** **a**, A coronal view of a whole-body CT scan image with regions of each body part (annotated in green); **b**, Representative CT images of 16 different body parts in axial view: L1=Brain, L2=Eye lens, L3=Nose, L4=Salivary gland, L5=Thyroid, L6=Upper lung, L7=Thymus, L8=Heart, L9=Chest, L10=Upper abdomen, L11=Lower abdomen, L12=Upper pelvis, L13=Lower pelvis, L14=Bladder, L15=Upper leg, L16=Lower leg.

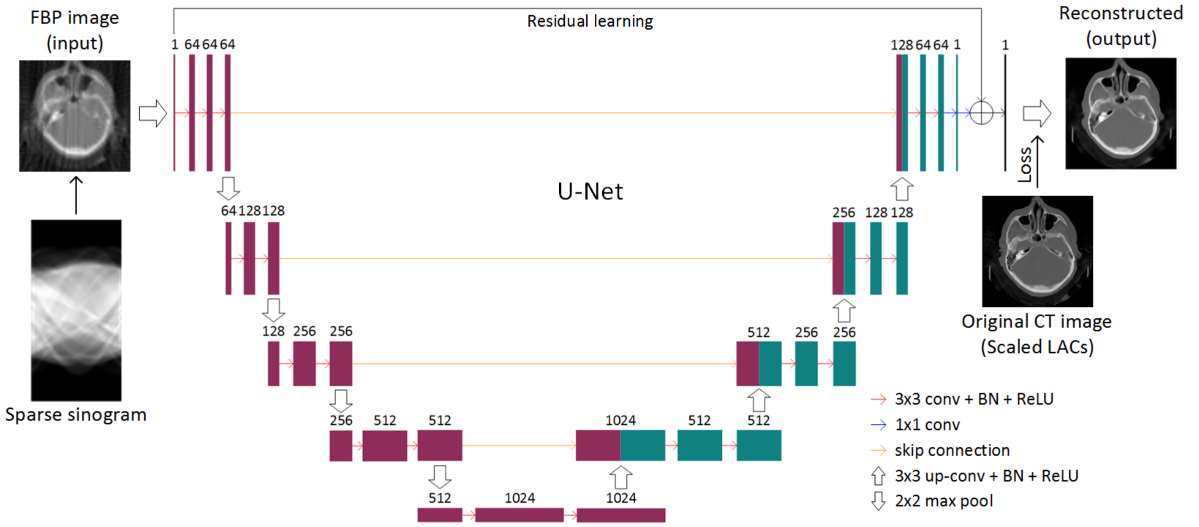


**Figure S2.** Network architecture of *FBPConvNet* for sparse image reconstruction proposed by Jin et al[8]. The *FBPConvNet* is a modified U-net which employs multilevel decomposition and multichannel filtering with a skip connection between input and output for residual learning. FBP, filtered backprojection; LAC, linear attenuation coefficients

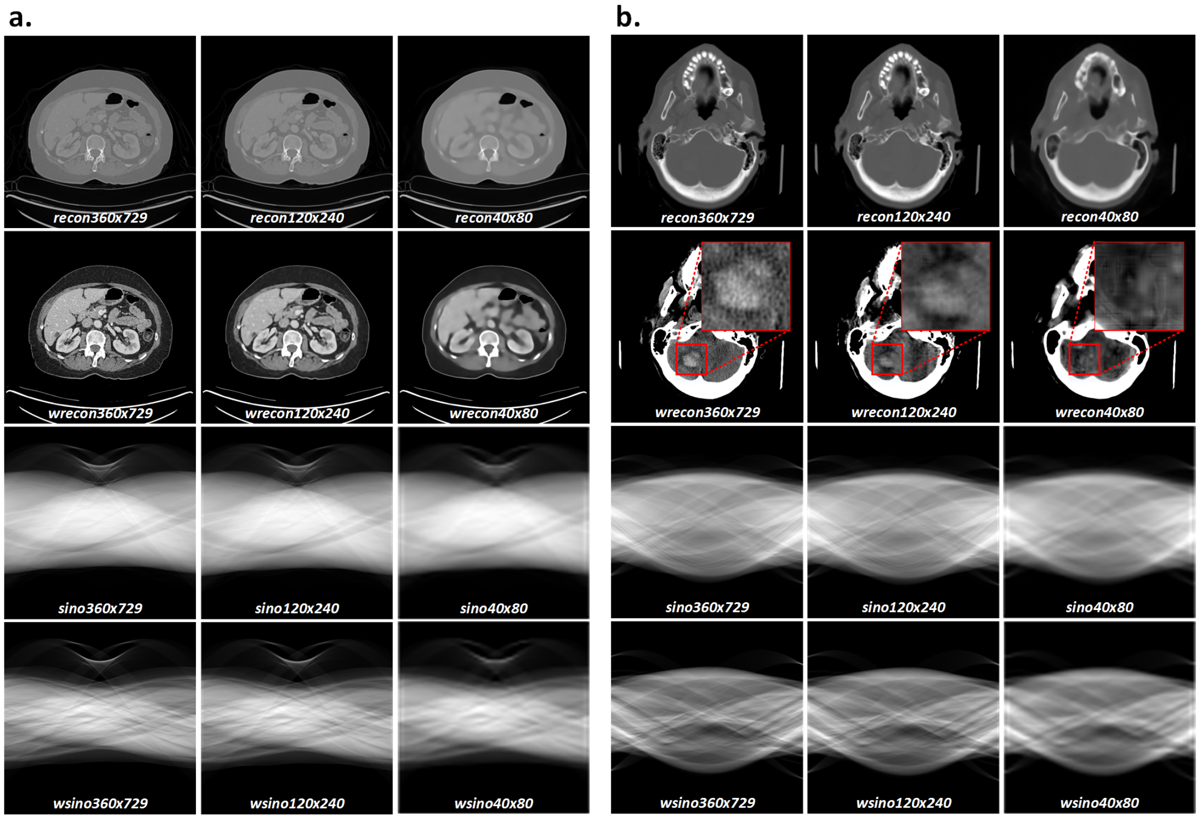


**Figure S3.** Example images for sinograms ('*sino360x729*', '*sino120x240*', '*sino40x80*') and reconstructed images ('*recon360x729*', '*recon120x240*', '*recon40x80*') for **a**, body part recognition and **b**, ICH detection tasks. Windowed reconstruction images were generated by applying abdomen window (window-level = 40 HU, window-width = 400 HU) for body part recognition and brain window (window-level = 50 HU, window-width = 100 HU) for ICH detection. All reconstructed images and sinograms are normalized to the same resolution for this figure.

| | Body part recognition | | ICH detection | |
|---|---|---|---|---|
| | FBP | FBPConvNet | FBP | FBPConvNet |
| *sino120x240* | 1155.4 ± 19.3 | 28.6 ± 6.2 | 1251.5 ± 35.6 | 26.8 ± 8.5 |
| *sino40x80* | 1147.2 ± 19.4 | 66.9 ± 16.6 | 1238.1 ± 34.2 | 66.1 ± 21.2 |

**Table S1**. RMSE computed on the validation dataset between scaled LACs converted from original CT images and reconstructed images ('*recon120x240*', '*recon40x80*') through FBP and *FBPConvNet* from sparse sinograms. RMSE values are expressed as mean ± standard deviation.
